# Supplementary figures and images for: Effects of artificially introduced Enterococcus faecalis strains in experimental necrotizing enterocolitis
Source: PLoS One. 2019 Nov 1;14(11):e0216762. doi: 10.1371/journal.pone.0216762 (PMC6824573; doi:10.1371/journal.pone.0216762)

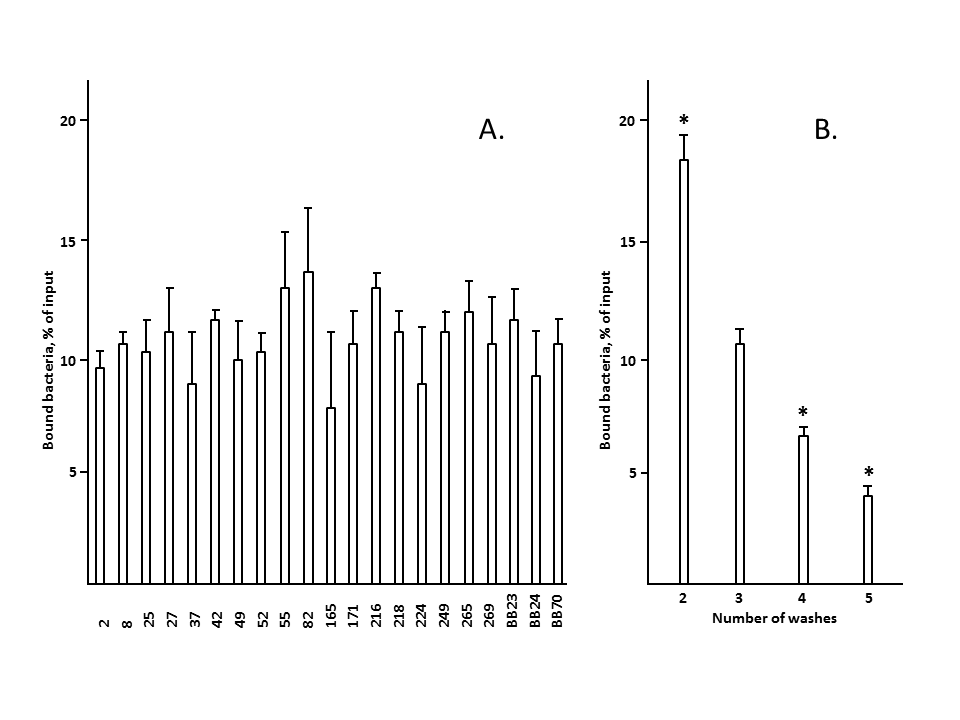

Supplement: S1 Fig — (TIF) [file pone.0216762.s001.tif]
